# Dynamically predicting comprehension difficulties through physiological data and intelligent wearables

**DOI:** 10.1038/s41598-024-63654-z

**Published:** 2024-06-13

**Authors:** Haytham Hijazi, Miguel Gomes, João Castelhano, Miguel Castelo-Branco, Isabel Praça, Paulo de Carvalho, Henrique Madeira

**Affiliations:** 1 CISUC, University of Coimbra, 3004-531 Coimbra, Portugal; 2 School of Engineering, Polytechnic of Porto (ISEP/IPP), 4249-015 Porto, Portugal; 3 ICNAS, University of Coimbra, Coimbra, 3000-548 Portugal; 4 ICNAS/CIBIT, University of Coimbra, Coimbra, 3000-548 Portugal

**Keywords:** Language comprehension, Machine learning, Biosensing, Eye-tracker, Wearable devices, Predictive markers, Cognitive neuroscience, Language, Reading, Data integration, Machine learning, Software

## Abstract

Comprehending digital content written in natural language online is vital for many aspects of life, including learning, professional tasks, and decision-making. However, facing comprehension difficulties can have negative consequences for learning outcomes, critical thinking skills, decision-making, error rate, and productivity. This paper introduces an innovative approach to predict comprehension difficulties at the local content level (e.g., paragraphs). Using affordable wearable devices, we acquire physiological responses non-intrusively from the autonomous nervous system, specifically pulse rate variability, and electrodermal activity. Additionally, we integrate data from a cost-effective eye-tracker. Our machine learning algorithms identify ’hotspots’ within the content and regions corresponding to a high cognitive load. These hotspots represent real-time predictors of comprehension difficulties. By integrating physiological data with contextual information (such as the levels of experience of individuals), our approach achieves an accuracy of 72.11% ± 2.21, a precision of 0.77, a recall of 0.70, and an f1 score of 0.73. This study opens possibilities for developing intelligent, cognitive-aware interfaces. Such interfaces can provide immediate contextual support, mitigating comprehension challenges within content. Whether through translation, content generation, or content summarization using available Large Language Models, this approach has the potential to enhance language comprehension.

## Introduction

The body of knowledge is filled with abstract concepts communicated through natural languages, prominently observed in written texts. The ubiquity of digital platforms such as smartphones, tablets, and PCs has transformed how written content is accessed, causing readers characterized by high digital literacy but insufficient reading comprehension^1^. Reading for comprehension in the digital environment is a daily practice that involves intricate perceptual, mental, and motor operations necessary to understand language at various levels. Comprehension assessment is a complex task, often compared to the enormity of general thinking processes^2^.

The increasing use of online content for foreign language learning, especially English, the de facto language of communication, introduces challenges such as lexical complexities and convoluted phrases that impede comprehension. Poor language comprehension in digital media may have significant consequences, such as learning impairment, reduced critical thinking, reduced quality of work, and higher error rates at work.

Readers may resort to revisiting challenging regions or employing external resources, such as translators, Artificial Intelligence (AI) generative tools, and search engines, which disrupt the reading experience and engagement. To address this challenge, we propose an intelligent approach capable of predicting and identifying comprehension difficulties in digital material at a local level of content, such as paragraphs. The approach relies on capturing cognitive load changes by measuring real-time physiological responses through biofeedback wearable devices and utilizing AI to predict when and where comprehension difficulty occurs.

This paper introduces and evaluates the approach. We utilize physiological responses (biomarkers) from the Autonomic Nervous System (ANS), including Pulse Rate Variability (PRV) and Electrodermal Activity (EDA), captured using wearable devices such as smartwatches. The PRV is commonly measured using pulse wave signals obtained from photoplethysmography (PPG), which is a basic optical method that detects changes in blood volume in peripheral circulation. The PPG is accessible in wearables and smartwatches. On the other hand, EDA reflects the continuous changes in the electrical properties of the skin, which is also accessible in wearables and some smartwatches.

While reading, the cognitive load induced by comprehension difficulties manifests itself in central and autonomic nervous system activities and is detectable through low-intrusive biosensors (e.g., PPG and EDA sensors). Most key biosensors are now available in commercial wearables, bracelets, and smartwatches^3^. Additionally, to enable the approach to identify local difficulties in the content, an affordable desktop eye-tracker is integrated into the evaluation setup. We refer to these local difficulties as “hotspots,” which are regions in the content (e.g., paragraphs) associated with high cognitive load and potential comprehension difficulties.

Thus, this paper aims to answer the following questions:

1. Can physiological biomarkers like PRV and EDA recognize changes in cognitive load that correlate with comprehension during English reading tasks, particularly as a foreign language, at the paragraph level?
2. Can these physiological biomarkers, combined with low-cost eye-tracker data, identify English text content regions associated with comprehension difficulties experienced by individuals?
3. What is the best machine learning model to generalize different users’ behaviors in content comprehension during English reading tasks?

To address these questions, a controlled experiment was conducted with 40 Non-native English speakers participants from higher education and research institutions, encompassing various academic levels. The participants were provided with three English texts of varying complexity (simple, intermediate, and difficult) and equipped with an Empatica E4 wristband^4^ while using a Tobii 5L desktop eye tracker^5^ attached to a laptop. The participants were asked to carefully read the English texts to answer comprehension questions after each task. Based on comprehension question responses and a self-evaluation of the participants’ English level, we classified them as “standard” and “expert.” It should be noted that, for analysis purposes, we segmented the texts into semantically coherent regions with varying CEFR levels^6^. Furthermore, participants received a subjective assessment to express their perceived difficulty using the NASA-TLX questionnaire^7^. Importantly, the participants were asked to use “read” and “yellow” digital highlighters while reading the texts to annotate parts or text regions they felt were difficult or uncertain about, respectively. In other words, we involved our participants in the labeling process to minimize any uncertainty in the modeling phase.

Existing research on text comprehension assessment using objective methods has frequently depended on single modalities like eye-tracking or neuroimaging techniques such as electroencephalography (EEG), functional near-infrared spectroscopy (fNIRS), and functional magnetic resonance imaging (fMRI), which directly measure brain activity.

The practical limitations of these methods in everyday contexts led us to explore a more practical alternative: physiological responses obtained from lightweight sensors and commercial wearables. While previous studies focused on emotion recognition, engagement level assessment, and code comprehension, our work pioneers the use of these biomarkers to directly assess cognitive load associated with content comprehension in natural language contexts. However, cognitive load as a construct has been extensively explored in various studies that linked physiological biomarkers or responses to cognitive load changes. For example, a very recent study reported in Sazuka et al.^8^ investigates the connection between physiological responses and cognitive load, with particular emphasis on heart rate variability (HRV) and electrodermal activity (EDA) as viable biomarkers of cognitive load from the autonomic nervous system. The study utilized features derived from HRV and EDA, such as the Root Mean Square of the Successive Differences (RMSSD) for HRV and Skin Conductance Rate (SCR) for EDA. However, this study used the three-back and zero-back mental tasks as stimuli and did not explore other mental tasks, like reading and comprehension.

Another important study established a general framework for cognitive load estimation found in Ahmad et al.^9^. In this study, the authors suggest a method for non-invasively monitoring physiological data from the heart and eyes to measure cognitive load in real-time. Using a combination of classifiers, such as Random Forest and Naive Bayes, the researchers were able to predict low, medium, and high levels of cognitive load through the use of activities that produced varying degrees of cognitive load without mentioning the nature of the activity. It was shown that critical features like mean pupil diameter change and blinking rate were important in accurately predicting the degrees of cognitive load.

Likewise, in a study by Vanneste et al.^10^, the authors aimed to investigate the relationship between cognitive load and physiological responses using a multimodal approach. The features they used to measure cognitive load included the duration and rate of the skin conductance response, the blink rate of the eye, in addition to the alpha power, the alpha peak frequency from the brain activity. These features were monitored through EDA, electroencephalography (EEG), and electrooculography (EOG). Again, the stimuli and tasks that were used in this study was the tangram puzzle game to induce varying levels of complexity.

As we can observe from previous studies that measured cognitive load through physiological responses, the focus has not been on reading comprehension, particularly on natural language comprehension at local levels

In light of this, this paper contributes to the field in several ways:

- This study is the first attempt, to the best of our knowledge, to employ lightweight biosensors and wearables in conjunction with a low-cost eye tracker to assess comprehension levels in natural language, providing a vital use case for these technologies.
- It extends content comprehension analysis beyond the task level, predicting comprehension at local regions of text through the synchronous analysis of physiological biomarkers and eye-gaze information.
- The study presents an evaluation setup approximating a realistic prototype, offering potential applications in learning, educational, and professional environments.

The structure of the paper is organized as follows. The next section provides the results of the approach evaluation. The paper then presents the discussion and implications of this approach. Finally, the paper presents the methods and the setup and configurations of the controlled experiments.

## Results

**Figure 1:**
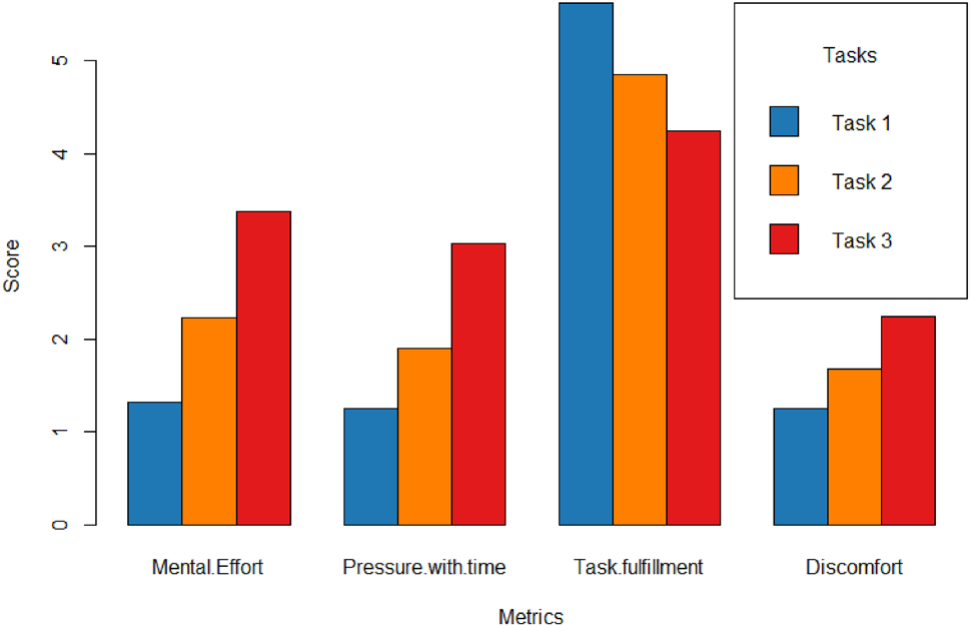
NASA-TLX results.

The results of our approach evaluation can be grouped into three levels. First, we present the participants’ NASA-TLX subjective assessment of the reading tasks to get an essence of the complexity of these tasks (Fig. 1), and we show the actual comprehension of participants’ performance based on the comprehension questions. Second, we show the changes in cognitive load at the levels of the comprehension task and the content region, identifying significant PRV, EDA and eye tracking characteristics (RQ1). Finally, we provide the results of the machine learning predictive model in inferring content comprehension at the task and content region level (RQ2 and RQ3). These results show discriminating PRV, EDA, eye tracking, and contextual features in different modalities with various feature selection and classification methods.

### NASA-TLX performance and task level analysis

Figure 1 shows the NASA-TLX results. The figure reveals that Task 1 was perceived as the easiest task with the least pressure and discomfort, while also resulting in the highest feeling of fulfillment.

In contrast, Task 3 was perceived as the most mentally demanding task with the highest pressure and discomfort, while also resulting in the least feeling of task fulfillment. Task 2 was rated between Task 1 and Task 3 in terms of perceived difficulty. The NASA-TLX results align with our description of the task characteristics presented in Table 1.

**Table 1:** Task Information.

Task	Level (CEFR)	Expected difficulty	Number of regions	Flesch-Kincaid score
Text 1	A2	Simple	2	61.9
Text 2	B2	Intermediate	3	58.7
Text 3	C2	Difficult	2	44.2

**Figure 2:**
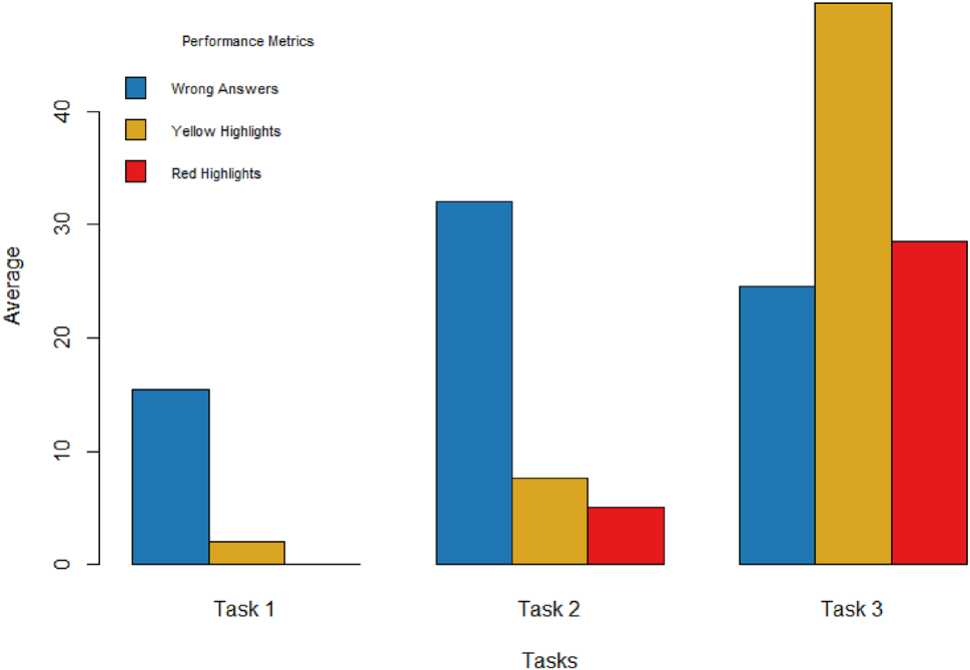
Participants’ comprehension performance at the task level.

To confirm that with the participants’ actual comprehension performance at the task level, Fig. 2 shows the performance reflected by the average of incorrect answers to comprehension questions, providing a complementary perspective on participants’ task engagement and comprehension ability. Figure 2 also depicts the number of red and yellow highlights per task, which reflect the difficulty and uncertainty encountered in the content, respectively.

Based on Fig. 2, Task 1 had the fewest average wrong answers and the lowest average number of yellow and red highlights, while Task 2 had the highest average wrong answers and Task 3 had the highest average yellow and red highlights.

#### Local level analysis

**Figure 3:**
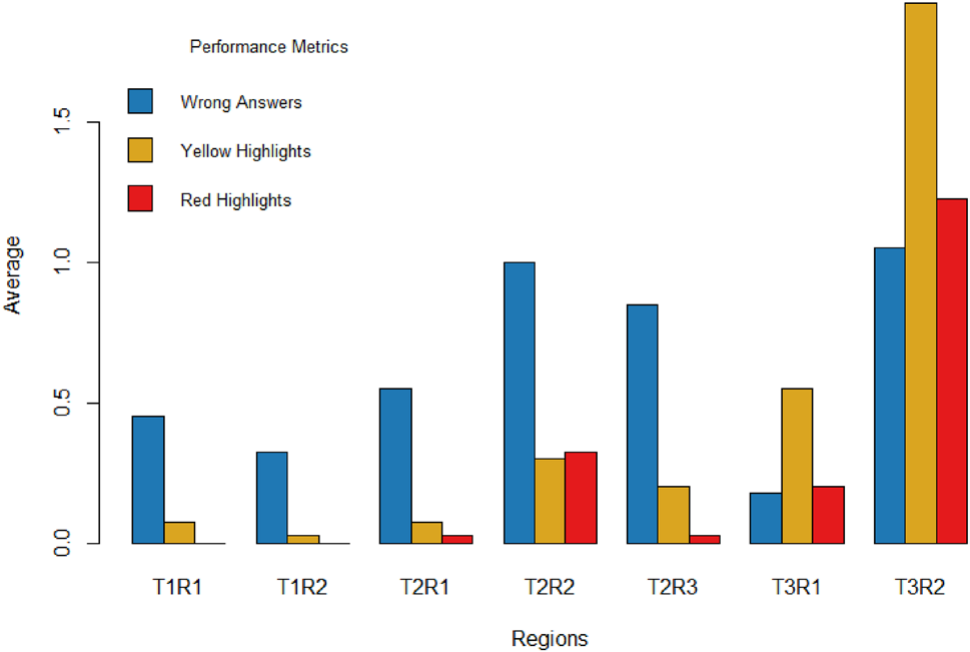
Participants’ comprehension performance at the content region level.

Digging deeper into the data, Fig. 3 shows the average wrong answers, yellow highlights, and red highlights at the local level for each task (i.e., text). These findings align with the overall results of the NASA-TLX. In Fig. 3, we can see that Region 2 of Task 3 (T3R2) had the highest values for incorrect answers, yellow highlights, and red highlights, indicating that this section could be the most challenging for the average volunteer in terms of comprehension difficulty.

Overall, these results demonstrate the relative performance and difficulty of each task and provide insight into the specific areas where participants may have struggled.

After getting the essence of participants’ performance at task and local levels, we address RQ1. However, it is essential to get a notion of the extracted physiological features, which are shown in Table 2.

**Table 2:** Overview of features.

Domain	Feature name	Overview
PRV (time domain)	Mean HR	Average heart rate
	SDSD	Standard deviation of the difference between successive RR intervals in ms
	RMSSD	Root mean square of the difference between successive RR intervals
	SDNN	Standard deviation of RR intervals in ms
PRV (non-linear)	SD12	Ratio between the minor and major axis of the PointcarÃ© beat to beat time
PRV (Frequency domain)	LF/HF	Ratio between low and High frequencies
EDA	SCL	Tonic component of the EDA signal
	SCR	Phasic component of the EDA signal
	EDAPeakrate	Ratio of the number of SCR values divided by the data time
Eye-gaze (eye-tracker)	TotalTimeSeconds	Number of seconds spent looking at a region represents the reading time
	RevisitsNumber	Number of revisits corresponds to the frequency with which the individual looks back

It’s worth noting that these features were obtained through two methods: domain knowledge, which we refer to as handcrafted, and a data-driven approach based on feature selection methods that will be discussed later in this paper.

We investigate the potential of physiological biomarkers, such as PRV and EDA, to detect cognitive load changes during English comprehension tasks, both at the overall task level and at the local level within specific regions of the text. The aim is to determine whether we can identify distinctive features that capture the complexity of comprehension tasks and their corresponding regions. The null hypothesis (H0) posits that there are no statistical dependencies between task complexities and the PRV/EDA features, while the alternative hypothesis (H1) suggests the presence of significant dependencies between task complexity and these physiological features. We conducted the Shapiro-Wilk test to assess the normality of the features, and the obtained p-value was less than 0.0001, indicating that the features are not normally distributed. Consequently, we used the Mann-Whitney U test (Wilcoxon rank-sum test) for all the features to compare their distributions between different task complexities.

**Figure 4:**
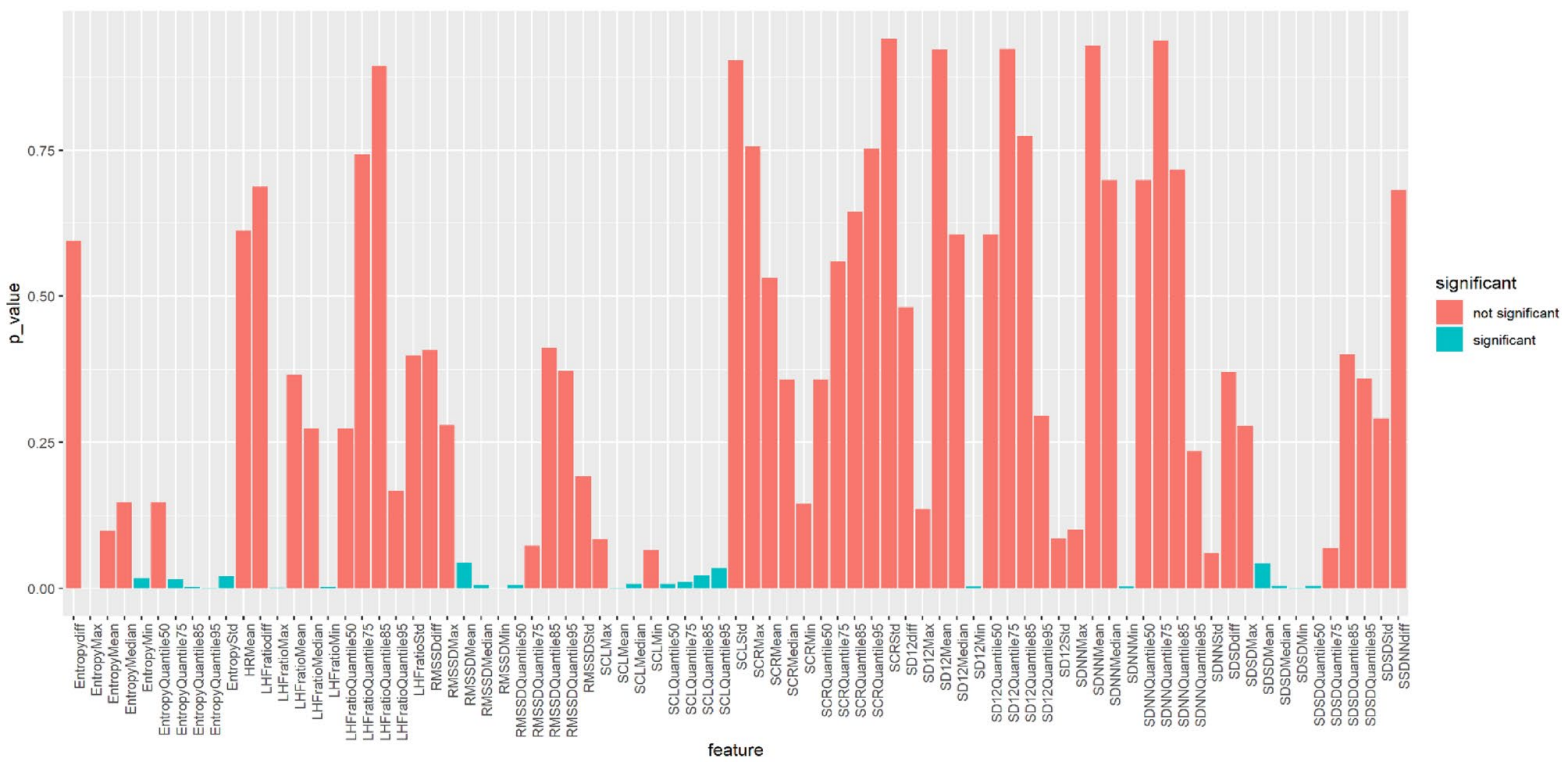
PRV and EDA significant and non-significant features: task level.

After performing the test, Fig. 4 shows the significant (the green bars) and non-significant (the red bars) features at the task level, indicating that both time and frequency domain PRV features show significant differences among tasks, including RMSSD, SDNN, SDSD, LF/HF ratio, and entropy features. EDA features exhibit significant differences among tasks as well, such as SCL mean, median, and minimum values, albeit to a lesser extent. Nonetheless, we could reject the null hypothesis (H0) for the features indicated by the green bar, showing that there is a significant dependence between the changes in these features and the reading task complexities.

**Figure 5:**
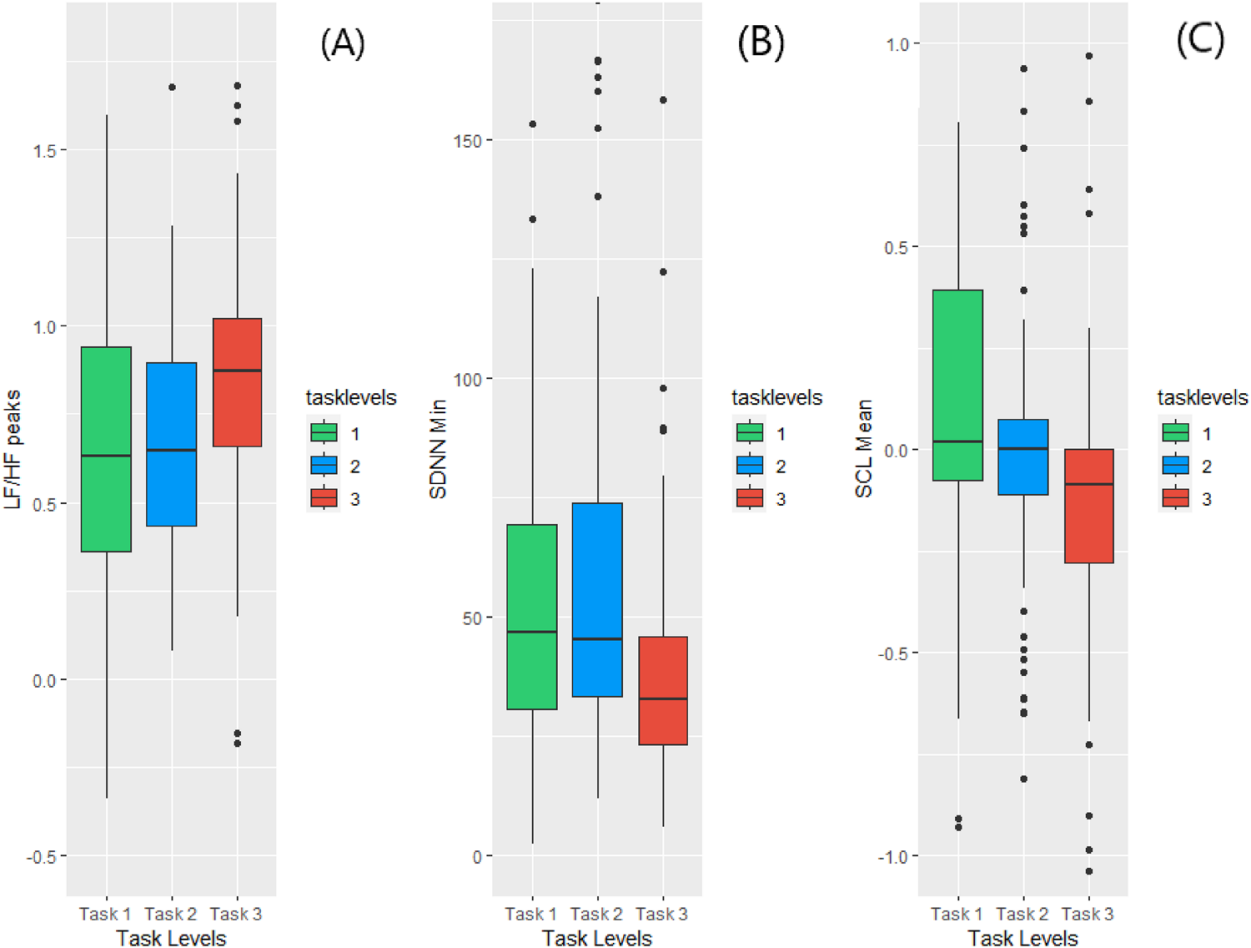
The change behavior of (**A**) LF/HF, (**B**) SDNN, (**C**) SCL with tasks.

In our observations, we found significant differences among tasks in the maximum peaks of the PRV frequency domain (e.g., LF/HF maximum), entropy features (e.g., entropy maximum), and minimum peaks of the PRV time domain (e.g., RMSSD minimum). However, it was unexpected that only the slow response part of EDA features, represented by the Skin Conductance Level (SCL) features (e.g., SCL Mean), showed significance. Figure 5A, B, and C show the behavior of significant features for PRV and EDA concerning task complexity. For example, Fig. 5A shows the PRV LF/HF (low frequency/high frequency) peaks feature for the three tasks (T1, T2, and T3).

The Kruskal-Wallis’s test and post hoc analysis with Bonferroni correction indicated no significant differences between T1 and T2, but there were significant differences between T2 and T3 (*p* = 0.0022) and between T1 and T3 (*p* = 0.0122).

The LF/HF represents the sympathovagal imbalance. Higher LF/HF indicates higher domination of the sympathetic system, which implies a higher mental workload in general^11,12^. Similar results were observed for other significant features, as shown in Fig. 5B and C

Figure 5B shows an opposite change in direction. The higher the complexity of the task, the less the SDNN becomes. A recent study^13^ shows that SDNN evokes lower values in higher knowledge-based tasks. The Kruskal-Wallis’s test and post hoc analysis with Bonferroni correction indicated no significant differences between T1 and T2, but there were significant differences between T2 and T3 (*p* = 0.0022) and between T1 and T3 (*p* = 0.0038). However, contrary to different studies, EDA increases with the cognitive state, especially the stress level. Our results reveal that, on average, SCL features (e.g., SCL mean) drop with the complexity of the reading task, as shown in Fig. 5C.

**Figure 6:**
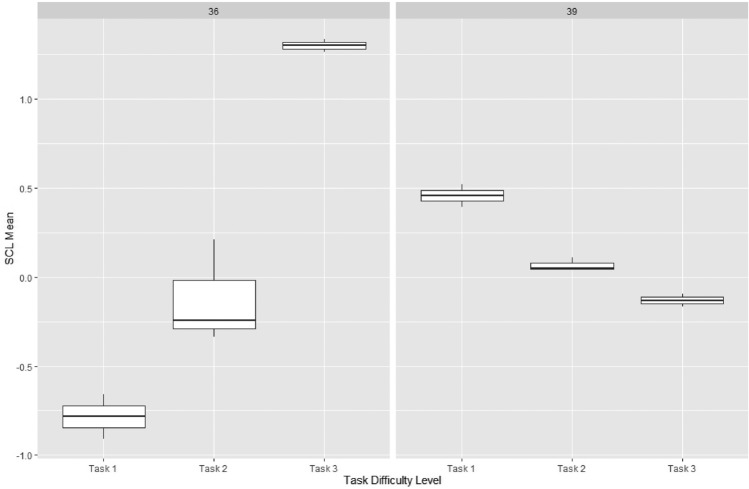
SCL subject dependency in tasks.

There are two possible interpretations of this result: a) when people are involved in a comprehension task and highly engaged, they might not feel stressed due to that full engagement with the content, which could increase with the task complexity; and b) studies^14,15^ demonstrate that EDA is highly reliant on subjects. For example, in the study reported here^14^, the authors show that with complex tasks, EDA means differences tend to react in an unexpected direction (i.e., similar to what we observed). To validate the latter argument, we show an example in Fig. 6. The example shows Participant 36 tends to have an increased SCL mean with the increased complexity of the task, whereas Participant 39 has an inverse tendency. We tested the significance through Wilcoxon between each pair of tasks, and also among the three tasks using Kruskal-Wallis. The results indicate that both participants showed significance between Task 1 and Task 3 (*p* = 0.0122) and Task 2 and Task 3 (*p* = 0.0022).

To gain more insights, we divided our participants into expert and standard groups based on their English proficiency level. We also incorporated their performance based on the number of incorrect answers they received on the comprehension questions. Participants with 0 incorrect answers in all questions were classified as ‘Good,’ those with 1 incorrect answer as ‘Fair,’ and those with 2-3 incorrect answers as ‘Poor.’ We visualized the mean levels of SCL based on these categories in Fig. 6.

In general, the SCL mean values tended to be higher in standard participants than in experts, particularly for the more challenging tasks (i.e., tasks 2 and 3) and in cases where they performed poorly on the comprehension assessment. We performed a Wilcoxon Signed Rank test to compare the SCL levels of different groups within each task, and the results are presented in Table 3.

**Table 3:** P-values for tasks comparing Expert and Standard conditions.

Tasks	*p* value
Task 1 (Expert vs. Standard)	0.04840
Task 2 (Expert vs. Standard)	0.00003
Task 3 (Expert vs. Standard)	0.10098

Since our study focuses on predicting content comprehension difficulties at the lodal level, we conducted an analysis to identify significant features from PRV and EDA data at this level. The following results in Table 4 display the significant features and their corresponding p-values, as determined by the Wilcoxon test.

**Table 4:** Significant PRV and EDA features at the local Level of Content.

Feature	*P* value
SDSDMin	0.001952
SDSDMedian	0.014215
RMSSDMin	0.000705
RMSSDMedian	0.022271
LHFratioMax	0.000613
LHFratioMin	0.014564
SD12Min	0.002269
EntropyStd	0.047456
EntropyMax	0.000044
EntropyMin	0.006439
EntropyQuantile95	0.000233
SCLMean	0.006651

The number of significant features at the local or paragraph level is lower than at the task level, particularly from EDA measurements. This is expected, as the time frame at the regional level is relatively short, which limits the ability of many features to capture the proper stimulus response and show significance across the varying complexities of regions.

To holistically answer RQ1, we dig deeper into the local analysis to explore the change in the direction of a specific feature across regions. The feature was randomly chosen from Table 4, which is SD12 min. In Table 5, we show each region comparison using Kruskal-Wallis’s test and Wilcoxon pairwise comparisons with Bonferroni correction.

As we can see from Table 5, if we take Task 2, we notice that Region 1 and Region 2 show a significant difference in the behavior of the PRV SD12Min feature. Likewise, in Task 3, we notice that Region 1 and Region 2 show a significant difference in the behavior of the same feature, showing a drop in its value. Based on the comprehension performance of participants, Task 2 Region 2 was more demanding than Task 2 Region 1 and likewise between Task 3 Region 1 and Task 3 Region 2, which justifies the decrease in the PRV ultra-short feature SD12Min when jumping from Region 1 to Region 2 in both tasks.

1. Can physiological biomarkers like PRV and EDA recognize changes in cognitive load that correlate with comprehension during English reading tasks, particularly as a foreign language, at the paragraph level?

Based on the previous quantitative and qualitative analysis, and the thorough discussions, we can conclude that physiological biomarkers from PRV and EDA are effective in recognizing changes in cognitive load associated with English comprehension tasks, both at the task and local levels.

**Table 5:** Wilcoxon PRV SD12Min at the local level with directions.

Task/Region	Task/Region	*P* value	Change direction (increase $\uparrow$ decrease $\downarrow$)
Task 1 Region 1	Task 2 Region 2	0.0191	$\uparrow$
Task 1 Region 2	Task 3 Region 2	0.0001	$\downarrow$
Task 2 Region 2	Task 3 Region 2	0.0165	$\downarrow$
Task 2 Region 3	Task 3 Region 2	0.0203	$\downarrow$
Task 2 Region 1	Task 2 Region 2	0.0205	$\downarrow$
Task 1 Region 1	Task 3 Region 1	0.0267	$\downarrow$
Task 3 Region 1	Task 3 Region 2	0.0306	$\downarrow$
Task 2 Region1	Task 3 Region 1	0.0467	$\downarrow$
Task 2 Region 1	Task 3 Region 2	$2.633 \times 10^{-6}$	$\downarrow$
Task 1 Region 1	Task 3 Region 2	$8.336 \times 10^{-6}$	$\downarrow$

Our findings suggest that PRV features are better able to capture changes in cognitive load than EDA in several cases. These results provide important insights into the use of physiological biomarkers as a means of assessing cognitive load in English language comprehension tasks, which could have significant implications for building the machine learning model that predicts comprehension difficulties.

Following the identification of cognitive load changes reflected by PRV and EDA, the present analysis result addresses the question of predicting comprehension difficulty. Given the complexity of comprehension as a mental process, which is not a linear function of cognitive load, the study explored different modalities of features. This included PRV and EDA standalone, a combination of PRV and EDA, and a handcrafted feature space. The handcrafted feature space incorporated PRV and EDA features, as well as behavioral and contextual information, in an attempt to improve the accuracy of the comprehension estimation. These features are (a) reading time, (b) number of revisits, and (c) experience level of the participant.

To avoid model bias and overfitting, a special version of Leave-One-Out Cross-Validation called Leave-One-Subject-Out Cross-Validation was employed during model evaluation and tuning.

#### Machine learning model performance

The performance of the model, comprising different modalities, feature selection methods, and classifiers, was evaluated and is presented in Tables 6, 7, 8, and 9.

**Table 6:** Performance metrics for different modalities and classifiers with backward feature selection.

Modality	PRV	EDA						
Classifier	Accuracy	Precision	Recall	F1	Accuracy	Precision	Recall	F1
LR	$54.33\%\pm 0.13$	0.54	0.55	0.54	$55.11\%\pm 0.33$	0.52	0.55	0.53
LDA	$52.01\%\pm 0.06$	0.57	0.52	0.54	$53.01\%\pm 1.51$	0.51	0.59	0.55
GNB	$61.45\%\pm 0.34$	0.55	0.53	0.54	$59.89\%\pm 1.23$	0.55	0.55	0.55

**Table 7:** Performance metrics for different modalities and classifiers with backward feature selection (Part 2).

Modality	PRV+EDA	Handcrafted features						
Classifier	Accuracy	Precision	Recall	F1	Accuracy	Precision	Recall	F1
LR	$57.28\%\pm 0.01$	0.58	0.69	0.63	$61.19\%\pm 0.21$	0.62	0.68	0.65
LDA	$58.44\%\pm 0.13$	0.60	0.58	0.59	$62.74\%\pm 0.84$	0.56	0.67	0.61
GNB	$57.78\%\pm 0.09$	0.60	0.60	0.60	$67.13\%\pm 0.83$	0.61	0.66	0.63

**Table 8:** Performance metrics for different modalities and classifiers with forward feature selection.

Modality	PRV	EDA						
Classifier	Accuracy	Precision	Recall	F1	Accuracy	Precision	Recall	F1
LR	$52.74\%\pm 0.56$	0.54	0.55	0.54	$66.00\%\pm 1.21$	0.55	0.66	0.60
LDA	$58.32\%\pm 0.08$	0.56	0.64	0.60	$56.75\%\pm 2.22$	0.53	0.56	0.54
GNB	$54.10\%\pm 0.41$	0.55	0.61	0.58	$55.39\%\pm 1.89$	0.58	0.64	0.61

**Table 9:** Performance metrics for different modalities and classifiers with forward feature selection (Part 2).

Modality	PRV+EDA	Handcrafted features						
Classifier	Accuracy	Precision	Recall	F1	Accuracy	Precision	Recall	F1
LR	$63.34\%\pm 2.21$	0.55	0.53	0.54	$72.11\%\pm 2.21$	0.77	0.70	0.73
LDA	$59.44\%\pm 0.69$	0.56	0.62	0.59	$67.22\%\pm 2.0$	0.64	0.67	0.65
GNB	$56.89\%\pm 0.78$	0.58	0.55	0.56	$67.56\%\pm 3.23$	0.66	0.67	0.66

The results demonstrate the relative effectiveness of the various modalities, feature selection methods, and classifiers used.

According to the findings of the present study, predicting content comprehension difficulty at the local level solely based on PRV or EDA physiological measurements is not recommended, as the performance is unsatisfactory. In contrast, when PRV and EDA measurements are fused, the precision and recall metrics show relatively better results. This could be because EDA enables us to distinguish cognitive stress from emotional stress. A handcrafted feature space consisting of selected physiological features from both forward and backward techniques, eye-tracking measures such as reading time and the number of revisits, and the experience level of participants was found to be the most effective approach for predicting content comprehension difficulty at the local level. The backward feature selection model yielded the most promising results, with logistic regression being the best-performing classifier in terms of accuracy, precision, recall, and f1-score metrics. Some of the top selected features from this technique are LF/HF (mean), RMSSD, SCL Mean, Reading Time, and SD12.

These findings have important implications for developing models to accurately predict content comprehension difficulty in non-native English speakers at the local level of English content. By combining multiple sources of information, including physiological and behavioral measures, as well as contextual factors such as experience level, more accurate predictions can be made.

RQ2) Can these physiological biomarkers, in conjunction with low-cost eye-tracker data, identify English content regions associated with comprehension difficulties experienced by individuals?

Based on the results presented in Tables 5, 6, 7 and 8, it is clear that wearable, unobtrusive devices that measure PRV and EDA, in conjunction with eye-tracking technology, can be used to successfully predict comprehension difficulties with good accuracy, precision, recall, and f-score. The inclusion of eye-tracking data in the model provided two valuable insights: (1) the specific region of the content where the individual is focusing their attention, and (2) eye-gaze features, such as reading time and the number of revisits to a particular content region.

RQ3) What is the best machine learning model to generalize different users’ behaviors in content comprehension?

Based on the performance results in Tables 5, 6, 7 and 8, the best results were achieved when combining both physiological and non-physiological features, such as behavioral and contextual information (i.e., handcrafted features). In terms of the feature selection method, the backward selection method was found to be the most effective, as it identifies and removes irrelevant features based on model results, thereby improving the model’s generalizability. It is worth noting, however, that this method can be computationally expensive. Finally, Logistic Regression was identified as the most effective classifier for the model. Logistic Regression is known for its simplicity and robustness to noise in the input space and for providing interpretable results, which can be important for understanding the factors that contribute to comprehension difficulty.

## Limitations, conclusions, and future directions

The approach proposed in this paper has demonstrated reasonable performance, but it still has a few limitations that explain why it could not achieve higher accuracy and precision. First, although this approach utilized a multimodal feature space, including biosignals and non-biosignals features, language comprehension, as a mental process, entails various and complex variables, such as mental states, emotions, linguistic features, prior knowledge, language decoding, mental representation of the text, and many others^16^. Thus, within the scope of our work, more contextual and complex variables, such as emotions, which we can assess using physiological signals that we presented in this paper, can be fused to improve accuracy and precision. Appropriate stimuli and well-planned experiments to train the machine learning model appropriately can help achieve this. Second, the time constraints of the controlled experiments limited the material (i.e, stimuli) that we could employ in this study. Longer, diverse texts that span a wide range of subjects and linguistic characteristics are needed to assess language comprehension (e.g., Wikipedia material). This limitation threatens the generalizability of the approach and may affect its precision. Third, even though 40 volunteers is a sizable cohort, it is important to recognize that their backgrounds and experiences are not very diverse. This limited focus may jeopardize our findings’ wider applicability to a wide range of users. Consequently, in order to guarantee a more thorough representation and improve the generalizability of our methodology, we are actively seeking the recruitment of extra volunteers from diverse backgrounds and areas of expertise.

One more important thing to mention when it comes to limitations is the setup’s accessibility and financial implications. Even with the use of wearables and coste effective eye-tracking technology rather than heavy and costly sensors, it is difficult to claim that all facilities and businesses can easily afford this kind of setting. As a result, our current efforts are focused on examining the viability of utilizing a webcam-based eye-tracker^17^ and using it to analyze facial expressions as well as pupilometry and gaze, which could result in substantial cost and setup time savings.

Despite these limitations, the study produced a number of important findings. In this study, we address the challenges of language comprehension difficulties by proposing a new technology that uses machine learning techniques, wearable devices and inexpensive eye tracking solutions. We conducted a controlled experiment involving 40 non-native English-speaking participants, equipping them with Empatica e4 wristbands and eye-tracking devices. The participants were instructed to read English texts of varying complexity and answer comprehension questions.

Our findings indicate that wearable biomarkers, specifically Pulse Rate Variability (PRV) and Electrodermal Activity (EDA), combined with eye-tracking data, can effectively identify content regions (hotspots) associated with comprehension difficulties. The accuracy, precision, recall, and f1-score achieved by our approach were reasonable, demonstrating the potential of wearable technologies and low-cost eye-tracking for comprehension assessment in learning and work environments.

To answer our research questions, we confirmed that physiological biomarkers such as PRV and EDA can recognize changes in cognitive load that correlate with English comprehension tasks at the paragraph level. Furthermore, when combined with low-cost eye-tracker data, these biomarkers can identify English content regions where individuals experience comprehension difficulties. We found that Logistic Regression when fed with biomarkers and handcrafted features, representing contextual and behavioral features, such as reading time, the number of revisits paid by an individual to a given content region, and experience level, offer the best accuracy, precision, recall, and f1-score.

We found that PRV features, including LF/HF ratio, SDNN, SD12, and RMSSD, exhibited changes that correlated with task complexity, cognitive load, and comprehension level. The higher LF/HF ratio observed with increasing task complexity suggests a higher mental workload and sympathovagal imbalance. On the contrary, the lower values of SDNN and RMSSD associated with higher knowledge-based tasks indicate a different aspect of the activity of the autonomic nervous system, indicating the cognitive demands of comprehension.

Our work unveiled unexpected trends in EDA behavior, particularly the decrease in SCL mean with increasing task complexity. These findings challenge previous research suggesting that EDA increases with cognitive load and stress. The observed decrease in EDA may indicate that highly engaged participants experience reduced stress and increased task immersion during comprehension tasks. However, the intra-variability in EDA responses highlights the importance of considering subject-specific characteristics when interpreting these findings.

Therefore, As we move forward, we understand that we must improve our approach even more in order to better account for individual variances. We intend to use a longitudinal strategy in our future study, which will enable the collection of a higher volume of individual physiological responses at various stages and times of the day. By taking a longer view, we should be able to better understand individual differences and increase the accuracy of our predictions. Furthermore, the methodology might be enhanced with concept drift approaches to detect changes in statistical properties and to adapt the models to these new contexts.

As a future direction, to address the challenge of generalizability, involving a more diverse population from various backgrounds will enable us to (i) identify user profiles and (ii) develop user-specific models to cater to each profile, thereby achieving a higher level of model personalization. Furthermore, these profile-specific models could be implemented using “transfer learning approaches” as the foundation for genuinely personalized models, integrating user-specific data collected by the system.

These findings have essential implications for understanding cognitive load and comprehension difficulties in various domains. Using PRV and EDA as physiological biomarkers, along with contextual information and eye behavior, our approach provides an objective and intelligent assessment of comprehension and cognitive load. This biofeedback approach can inform the development of personalized interventions, adaptive learning systems, and training programs that target specific areas of difficulty and optimize comprehension outcomes. In addition, it could target people with learning disorders (e.g., dyslexia).

Finally, this approach holds significant promise in the context of language learning, offering a valuable application. In the realm of learning a new language, both instructors and learners can leverage this technology to pinpoint particular sections, paragraphs, and linguistic constructs that pose challenges. As a result, the learning framework can be customized to cater to the unique requirements of each learner, ensuring a more effective and personalized language learning experience.

In summary, our study contributes to the growing body of research on the use of physiological biomarkers and wearables to assess cognitive load, engagement, and comprehension difficulties. By utilizing these biomarkers with contextual information, eye gaze behavior, and personal data, we can enhance comprehension assessment and improve learning, work quality, and decision making in various practical applications.

## Methods

**Figure 7:**
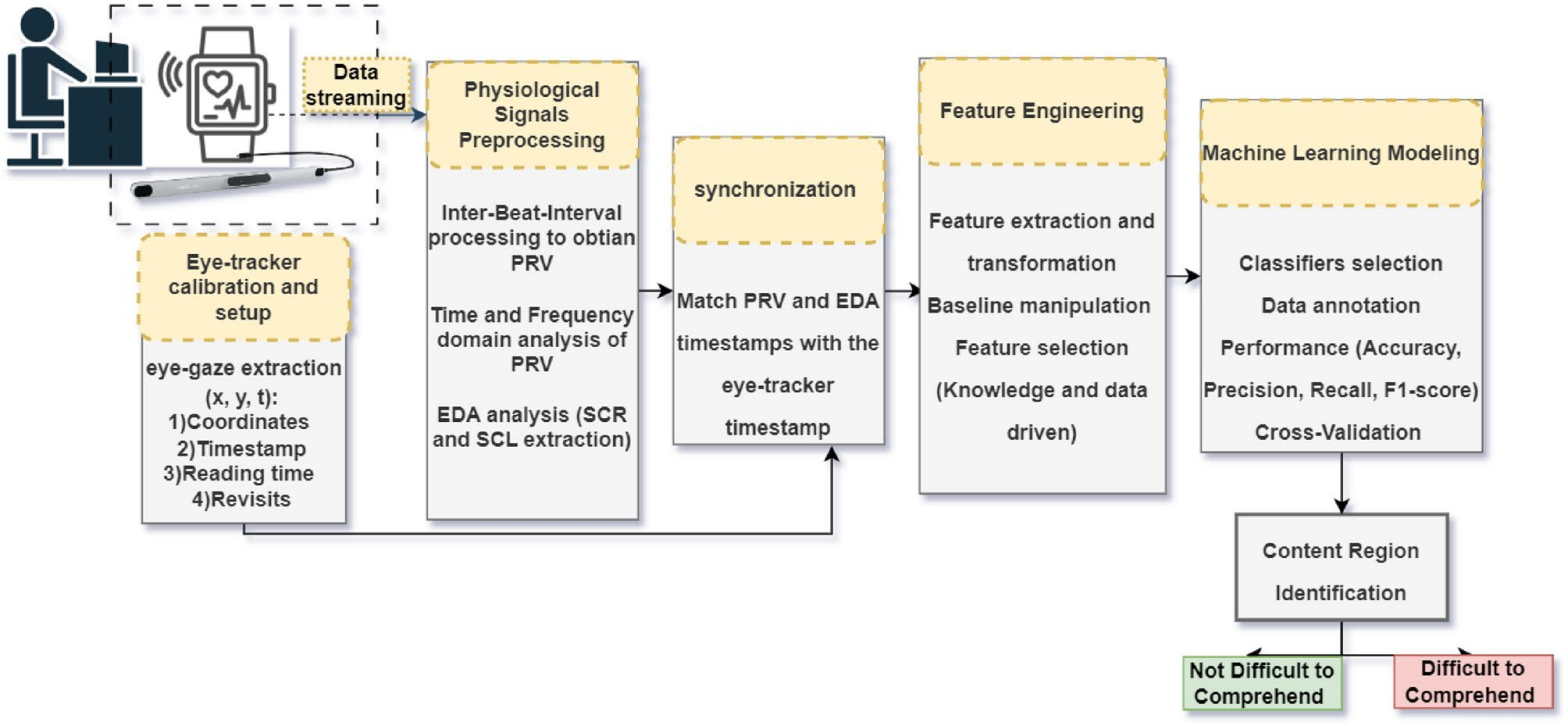
The proposed approach and its evaluation method.

Figure 7 shows the proposed approach, including the evaluation methodology.

The physiological data is acquired from users non-intrusively through wearable devices that offer seamless PRV and EDA continuous measurements. The mentioned physiological measurements are hypothesized to surrogate the cognitive load experienced by users while performing reading comprehension tasks. Synchronously, the eye-gaze data, which is represented by the user’s eye movements on the screen, is acquired through a desktop eye-tracker attached to the reading device (i.e., a laptop). The goal of using an eye-tracker is to identify content regions (hotspots) that are potentially associated with high cognitive load and thus might be difficult to comprehend. We used the desktop low-cost Tobii 5L eye-tracker, which works by observing the center of the pupil and the reflex of the cornea and creating the axis that gives the coordinates at which the user is looking. The synchronization between the wearable data and the eye-tracking data is achieved by the common timestamp between the two modalities. Furthermore, a new type of synchronization is performed to keep the page scroll of the content consistent with the timestamps from the measurements. The features from the physiological measurements are extracted per content region, which is specified by some content lines (e.g., paragraph).

However, before selecting the best features from both PRV, both time and frequency domain, and EDA, both skin conductance level (SCL)and skin conductance response (SCR), several pre-processing steps are followed for the physiological signals considered. Finally, the selected features, which will be illustrated in the next section, are fed into a machine-learning model to classify each content region as difficult to comprehend or not difficult.

To evaluate the accuracy of the proposed approach in predicting comprehension difficulties at the content region level, a controlled experiment was conducted. Further details are introduced in the next section.

### Experimental study

This section provides an overview of the controlled experimental study conducted to evaluate the proposed approach. It encompasses various aspects, starting with a description of the participants’ characteristics, followed by an explanation of the study protocol and the specific comprehension tasks administered during the study.

#### Participants

The proposed approach was evaluated through a controlled experiment involving 40 participants. The participant selection criteria included being a non-native English speaker, having a minimum B1 English level according to CEFR classification?, and not experiencing any cardiac disorders. Participants consisted of Bachelor, Master, and Ph.D. students, as well as lecturers and researchers from the University of Coimbra and ISEP in Porto. The gender distribution among the participants was 70% males and 30% females, with an average age of 28.1 years and a standard deviation of 11.2 years. Participants’ majors and specialties were categorized as follows: Languages (2.56%), Informatics Engineering (43.59%), Marketing (2.56%), Arts (2.56%), Biomedical Engineering (2.56%), Electronic Engineering (7.69%), Industrial Management Engineering (10.26%), Artificial Intelligence Engineering (10.26%), Computer Science (2.56%), Economics (2.56%), Mathematics (2.56%), and Medicine (2.56%). This diversity in the background could boost the model’s generalizability. Reading in English is required among all majors. The research study obtained ethical approval from the Ethical Commission of the Faculty of Medicine at the University of Coimbra, following the principles of the Declaration of Helsinki. All standard protocols for studies involving human subjects were strictly followed, including obtaining written informed consent from all participants. In addition, to ensure privacy, all data were anonymized. The anonymized raw data collected during the study can be made available to the research community on request by the authors.

It is important to mention that we emphasized that no data would be utilized for judgment or evaluation purposes, aiming to ensure that participants would feel relaxed during their performance in the experiment. The objective of this open and honest communication was to reduce the possibility that ambiguity or confusion would affect their performance or reactions. Additionally, minimizing confounding variables and guaranteeing the validity and reliability of the data obtained were made possible by giving clear instructions and revealing the evaluation criteria.

#### Reading comprehension tasks

The comprehension tasks consisted of English texts sourced from standard English as a Second Language (ESL) materials, including the IELTS, TOEFL, and TOEIC tests, and Cambridge English exams^18–20^. Table 1 in the previous section provided an overview of the main characteristics of each task. It is important to emphasize that this study primarily focuses on assessing comprehension difficulty at the language level, specifically in the context of foreign language comprehension, rather than evaluating the difficulty associated with the topics of the reading tasks. In other words, the content of the texts represents common knowledge known by all participants in the experiment, and possible difficulties in understanding such texts result from the fact that all participants are learning English as a foreign language (at different proficiency stages). Our goal is to isolate the source of comprehension difficulty, focusing solely on the language level. The segmentation of text into semantically coherent regions with varying CEFR levels^6^ has been undertaken with careful consideration in this study. The primary objective behind this segmentation is to conduct a meticulous analysis at a fine-grained level, specifically at the paragraph level. Nevertheless, it is essential to acknowledge that refining the granularity further may not yield precise measurements due to the inherent delay in physiological signal responses to stimuli. Consequently, opting for the granularity of a paragraph, or in more precise terms, a select set of language lines, emerges as an appropriate choice for our analytical framework.

The complexity of the texts was assessed using the Common European Framework of Reference for Languages (CEFR), which categorizes proficiency levels from A2 (basic user) to C2 (proficiency)^6^. Additionally, the regions within each task were defined based on semantic criteria and their complexity, as measured by the Flesch-Kincaid score^21^. This score indicates the difficulty of understanding an English passage by considering word length and sentence length. It ranges from 1 to 100, with higher scores representing greater readability. Text 3 aimed to introduce challenging vocabulary for non-native speakers, specifically vocabulary without Latin origins. The comprehension evaluation questions for the tasks were adapted from standard sources^18–20^. After careful task selection, the experimental protocol is introduced in the coming subsection.

#### Experimental protocol

**Figure 8:**
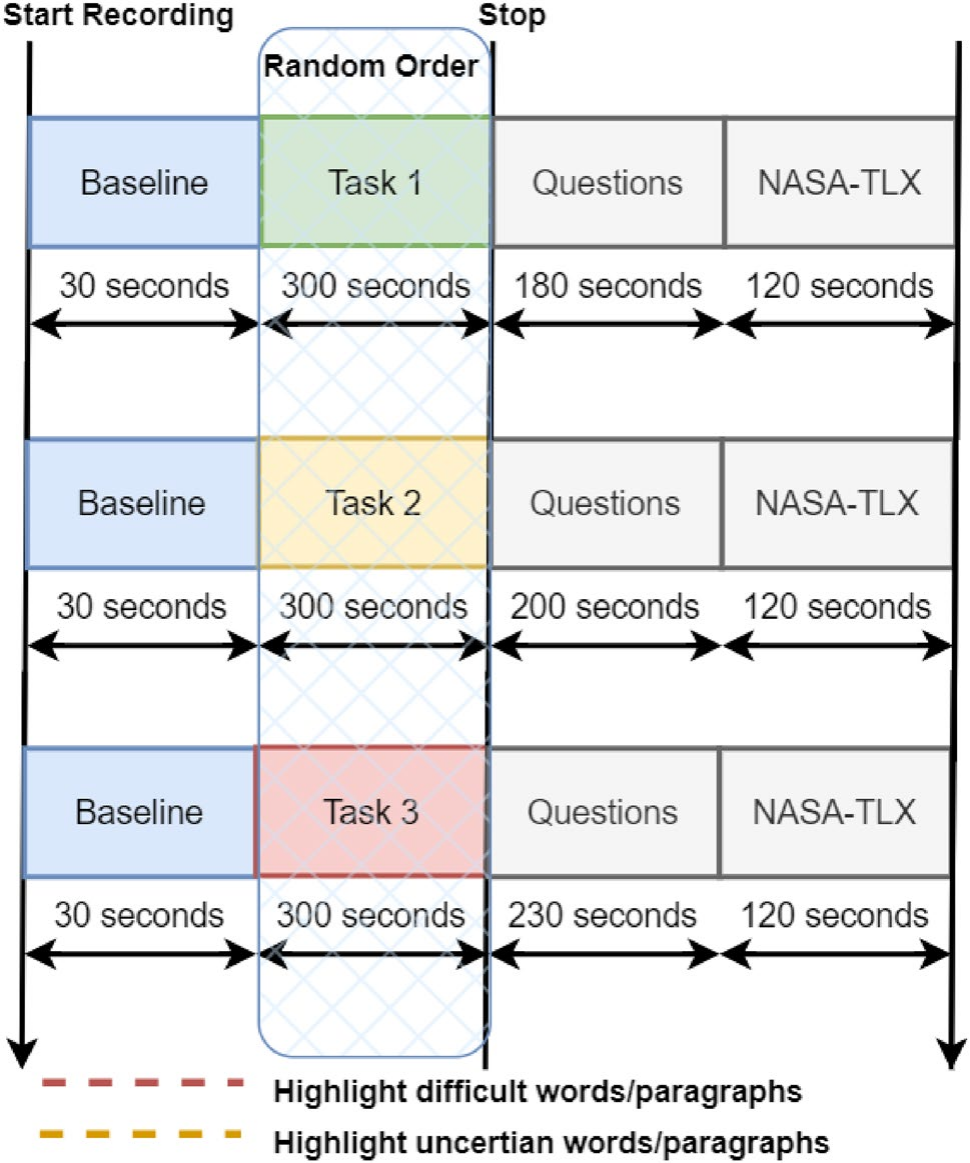
Experiment protocol.

The flow of the experiment and reading tasks is illustrated in Fig. 8. An experimental setup was developed as a web extension for the Chrome web browser to conduct the experiments. Upon starting the session, participants were presented with an information screen outlining the experiment’s goal, data acquisition, and privacy statement. After approval, physiological signal recording commenced, as depicted in Fig. 8 (i.e., start recording). To establish a baseline for physiological signals, a gray screen with a central cross appeared, promoting relaxation and reducing arousal and stress. After 30 seconds, the gray screen automatically disappeared, and the English reading comprehension tasks were displayed in random order to avoid bias. Participants were instructed to use the red and orange highlighters provided by the setup. The red highlighter was for annotating challenging content at the word, line, or paragraph level, while the yellow highlighter was for marking words, lines, or paragraphs they felt uncertain about. These annotations constituted the initial layer of ground-truth labels for the classifiers discussed later in this paper. Participants were advised to carefully read and comprehend the provided context and information, as comprehension questions would follow each task.

Each task was allotted a maximum time limit of 300 seconds, allowing participants the flexibility to proceed to the next task after a minimum of 180 seconds if they completed it. Subsequently, participants were presented with comprehension questions in the form of multiple-choice and true-or-false items, targeting specific text regions within the tasks. Following this, participants were introduced to adapted versions of the NASA-TLX survey^7^, which aimed to subjectively assess individuals’ impressions of reading comprehension tasks, including mental effort, time pressure, level of discomfort, and task fulfillment. The assessment scale ranged from 0 to 6, with 0 indicating the lowest score and 6 representing the highest score.

**Figure 9:**
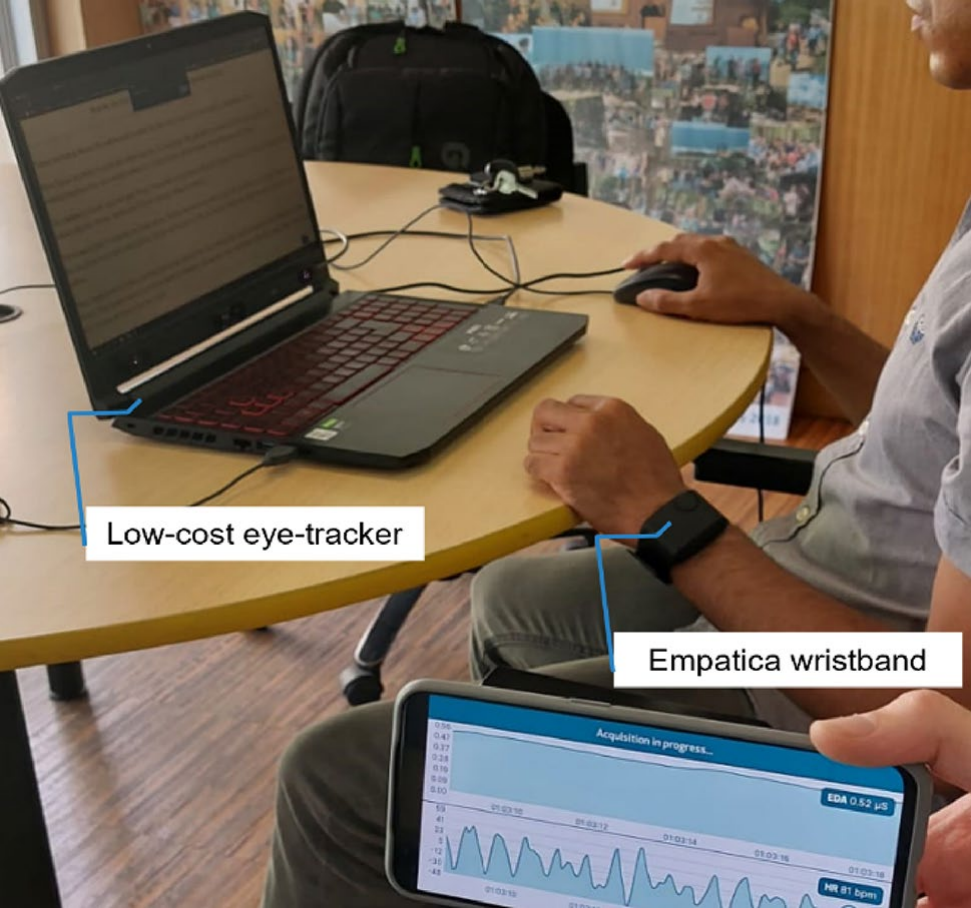
Experiment setup.

The authors of this study thoroughly evaluated the responses to the comprehension questions and the results obtained from the NASA-TLX assessment. It is important to highlight that the experiments were conducted in a natural environment, as depicted in Fig. 9, to replicate real-life scenarios of reading, comprehension, and engagement with content.

The figure illustrates a participant engaging in a typical daily life scenario of reading content on a laptop equipped with an affordable eye-tracker while wearing a wristband, which can be substituted with a smartwatch

#### Preprocessing and data analysis

**Figure 10:**
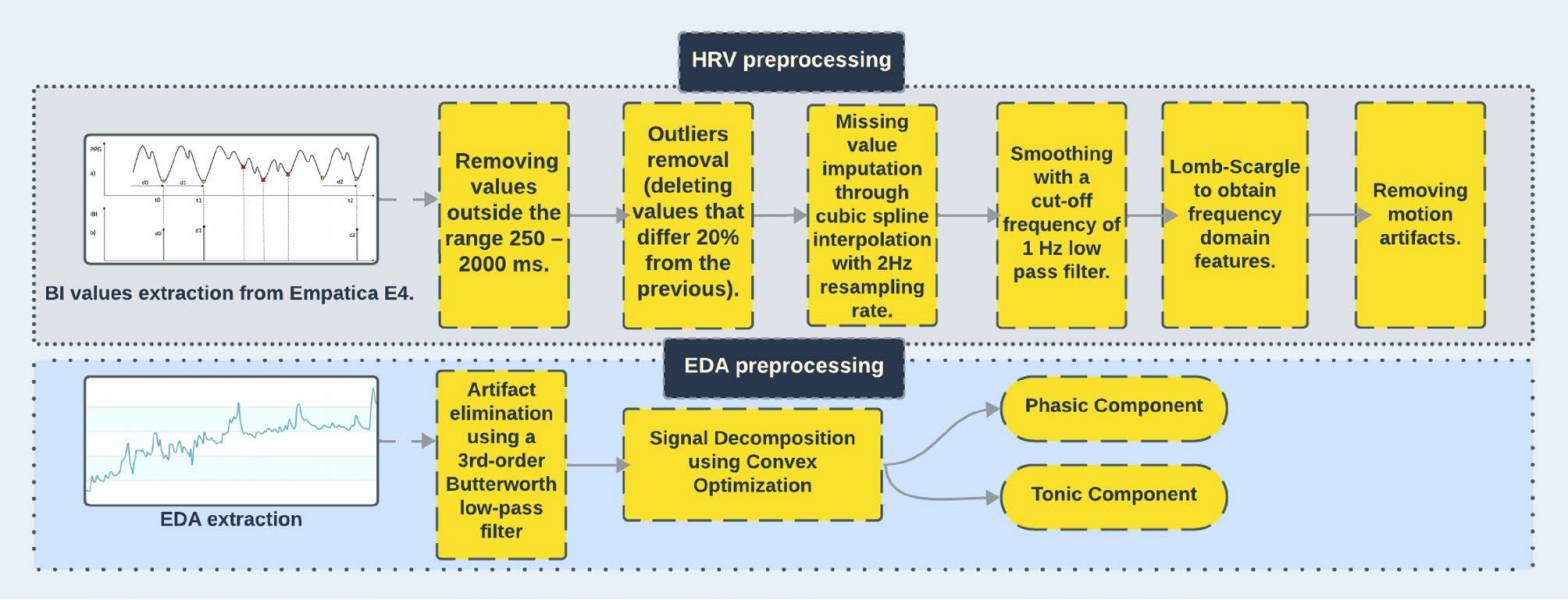
Preprocessing pipeline.

Figure 10 illustrates the preprocessing pipeline for PRV and EDA, which is based on established studies and best practices. While we referred to it as HRV preprocessing in the figure due to our use of Heart Rate Variability (HRV) guidelines, it is important to note that PRV alone cannot fully substitute for HRV.

The Empatica E4 device serves as the data source for PRV and EDA features. PRV is derived from the inter-beat interval (IBI) data, obtained by measuring the distance between peaks in the blood volume pressure (BVP) signal, specifically the diastolic points^22^.

To ensure data quality, we start by pre-processing the IBI values, as shown in Fig. 10. IBI outside the acceptable range of 250–2000 ms is removed^23^ (corresponding to a heart rate of 30 to 240 beats per minute). Additionally, outliers are identified and removed by deleting values that differ more than 20% from previous IBI values, following the PRV and Heart Rate Variability (HRV) guidelines^24^. It should be mentioned that none of the participants suffered from any known heart condition, such as Atrial Fibrillation, making this assumption plausible from a physiological point of view.

Frequency-domain features of PRV in the Lomb-Scargle method are computed by calculating the power or significance of different frequencies in the Lomb-Scargle periodogram. The Lomb-Scargle method uses least-squares fitting, which is employed to determine the amplitude and phase of sinusoidal components. To be more specific, the Lomb-Scargle periodogram achieves this by systematically adjusting the amplitude and phase parameters of a sinusoidal model to best match the time series data, which is the IBI data in this case^25^. Other PRV features were extracted, such as non-linear features, including the signal’s entropy and the ratio of the PoincarÃ© plot axis. The signal’s entropy captures the complexity and irregularity of PRV, making it detective to nonlinear dynamics in the autonomic nervous system. The ratio of the PoincarÃ© plot axis (SD1/SD2) provides a measure of the balance between short-term and long-term heart variability. This ratio has been shown to discriminate between different cognitive states, such as low and high cognitive states, more effectively than traditional PRV metrics^26^. Other classical time-domain features were also extracted per content region using overlapping sliding windows. Examples of such features include Mean Heart Rate (MeanHR), Standard Deviation of Successive Differences (SDSD), and Root Mean Square of Successive Differences (RMSDD). As for the EDA, the signal has two major meaningful components to indicate cognitive stress or emotional response. a) Skin conductance level (SCL) or the tonic component, which refers to the baseline level of the skin’s electrical conductivity; and b) Skin conductance response (SCR), which indexes the phasic changes in the skin’s electrical conductivity in response to stimuli. The EDA can be modeled as the sum of those components in addition to the white Gaussian noise, as shown in Formula 1^27^

1 $$\begin{aligned} \text {EDA} = \text {SCL} + \text {SCR} + \epsilon \end{aligned}$$

where $\epsilon$ is the additive white Gaussian noise.

The EDA is obtained from the Empatica E4 device with a uniform sample rate of 4Hz and undergoes noise removal and artifact elimination using a 3rd-order Butterworth low-pass filter with a cutoff frequency of 0.1Hz. The filter removes the negative values of SCR and SCL. The EDA data is then divided per content region. Separate processing is performed for EDA visits and revisits to account for their impact on signal decomposition. Signal decomposition into phasic and tonic components is achieved using a convex optimization approach algorithm^27^ to find the best combination of the three components that fit the original EDA signal, shown in Formula 3, utilizing the task baseline as the SCL part. Peaks of the SCR component are identified using a dynamic threshold response method^28^. SCR peaks reflect the collective activity of multiple sweat glands, each of which has a different activation threshold. The number of SCR peaks per time window is calculated to derive the corresponding phasic EDA features (e.g., SCR mean). Finally, for the eye-tracking features, we extracted visits and revisits, from which we computed the reading time. Visits are defined as the initial instances when the user directs their gaze towards specific content regions, while revisits refer to subsequent instances where the user looks at the same content regions again. To distinguish visits and revisits from smaller fixations and ensure sufficient time intervals for visits, we apply threshold values, following the approach employed in previous research^29,30^. The eye tracker data provides valuable information about the user’s gaze, including instances when the user looks away from the screen when the eye tracker temporarily loses track of the eye position due to obstructions or when the user moves out of the device’s field of vision. However, revisits are only counted when the user deliberately looks back to a previous region, and occurrences of looking outside the screen or temporary gaze loss are not considered revisits on their own. We use the timestamps of visits and revisits to segment the heart rate (HR) and electrodermal activity (EDA) data based on the content regions, enabling us to extract relevant features for each specific region.

After these processing steps, feature extraction was performed. However, to complete the modeling process, these features have to be labeled to use supervised machine learning. Therefore, we relied on three dimensions to quantify the comprehension level at each content region, namely: the number of wrong answers to comprehension questions denoted by $w$, the number of red highlights denoted by $r$, and the number of yellow highlights denoted by $y$.

The comprehension level function is defined as:

2 $$\begin{aligned} Comprehension\_Level(r, y, w) = {\left\{ \begin{array}{ll} 1 &{} \text {if } r> 0, y \ge 2, w > 0 \text { (difficult)} \\ 0 &{} \text {otherwise (not difficult)} \end{array}\right. } \end{aligned}$$

Our study identifies wrong answers and red highlights as indicators of increased difficulty in understanding content. We stress that yellow highlights should be considered less impactful than red highlights since uncertainty does not equate to difficulty encountered. Individual highlighting behaviors yield valuable insights: red highlights on a phrase or sequence imply a lack of understanding, while two or more yellow highlights indicate uncertainty and failure to comprehend the content. Detecting at least one wrong answer in a region suggests a lack of understanding. Assumption validity was confirmed through interviews with a subset of participants, addressing perceived difficulties and the use of red and yellow highlights. Additionally, the correlation between participants’ performance and their highlighting patterns further validates these assumptions.

#### Machine learning modeling

The prediction of an individual’s content comprehension using machine learning techniques based on physiological and behavioral data requires careful planning and implementation. We acknowledge the complex relationship that exists between cognitive load and comprehension difficulty in our study, which is essential to properly model the machine learning pipeline. According to definitions found in the literature^16^, the cognitive load is the amount of mental work or resources needed to complete an intellectual task effectively. Although previous studies have indicated that reducing cognitive load can improve comprehension, it is important to understand that variations in cognitive load may not always be directly associated with comprehension problems. Therefore, we implemented a careful strategy into our experimental design and modeling stage to mitigate this concern.

- In the experiment design, we asked volunteers to annotate content regions that are either difficult to comprehend or uncertain about their meaning. These annotations were utilized to partly train the machine learning algorithm. In other words, they contributed to formulating the labeling mechanism.
- Previous studies, such as Cain and Oakhill^31^, revealed that the amount of time spent reading during eye-tracking and the frequency of regressions (revisits) to a particular section of the text could be indicators of processing difficulties and possibly comprehension challenges.

The distribution of class labels is relatively balanced, with 44% of the data points belonging to class 1 and 56% to class 0.

After conducting feature extraction, feature selection, and comprehension modeling for labeling purposes, it is crucial to carefully select appropriate machine learning classification and predictive models. Additionally, the selection of an appropriate cross-validation technique must be thought out meticulously to avoid bias or overfitting issues. In our study, we used three classification models: Linear Discriminant Analysis (LDA), Logistic Regression, and Gaussian NaÏve Bayes.

First, LDA, a widely used technique for data analysis and classification, aims to identify linear combinations of features that maximize class separation. It establishes a separating hyperplane, represented by the formula:

3 $$\begin{aligned} f(x) = W^t X + B,\;\text { s.t. }\;x\;\text {belongs to class difficulty; if }\;f(x) > 0,\;\text {and belongs to non-difficulty; if }\;f(x) < 0 \end{aligned}$$

LDA maximizes class separability and minimizes intra-class variability using a hyperplane. This is a simple yet very robust approach since it tends to generalize due to its simplicity and effectiveness, especially in dealing with similar physiological signals^32^.

Second, Logistics Regression (LR) is an appropriate choice for classification given the binary output we have (e.g., difficulty vs. non-difficulty) with the continuous input features. Logistic Regression is known as simple, intrinsically explainable, and computationally efficient. LR is considered a technique for linear regression to operate in classification problems. The result of the classification is a value that lies between [0, 1], which is the probability $h(x)$ that the class of $x$ is either 0 or 1 after using the sigmoid function, as shown in the following formula:

4 $$\begin{aligned} f(y) = \frac{1}{1 + e^y}, \text { where } y = \beta _0 + \beta _1 x^1 + \beta _2 x^2 + \ldots + \beta _n x^n \end{aligned}$$

Where $x_1$ to $x_n$ are the features, and $\beta _0$ to $\beta _n$ are the weights.

Finally, the Gaussian Naive Bayes (GNB) classifier computes the likelihood of observing a given set of features provided a class label using the Gaussian probability density function. After that, it applies Bayes’ theorem to compute the posterior probability of each class given the observed features. The class with the highest posterior probability is assigned as the predicted class. We wanted to investigate the model while assuming there is a degree of independence between features, especially between physiological and non-physiological features.

To ensure the generalizability of the model and account for the subject independence of HRV and EDA signals, we employed the Leave-One-Subject-Out Cross-Validation (LOSOCV) technique. LOSOCV involves reserving one subject for evaluation while training the model on the remaining subjects. This process is repeated iteratively, with each subject serving as the evaluation set, and the results are averaged across all folds (subjects).

The rationale behind using LOSOCV in this context is that PRV and EDA signals exhibit both inter and intra-subject variability, meaning that the patterns and characteristics of these signals can differ significantly between individuals. By leaving out one subject at a time for evaluation, we ensure that the model is tested on unseen data from different individuals, providing a more realistic assessment of its generalization performance.

## Data Availability

To ensure the study’s applicability and the reproducibility of its results, both the code and the data utilized in this research are accessible via the following link: https://shorturl.at/cgzKR.
